# Toward a Taxonomy for Analyzing the Heart Rate as a Physiological Indicator of Posttraumatic Stress Disorder: Systematic Review and Development of a Framework

**DOI:** 10.2196/16654

**Published:** 2020-07-22

**Authors:** Mahnoosh Sadeghi, Farzan Sasangohar, Anthony D McDonald

**Affiliations:** 1 Department of Industrial and Systems Engineering Texas A&M University College Station, TX United States; 2 Center for Outcomes Research Houston Methodist Hospital Houston, TX United States

**Keywords:** heart rate, statistics, PTSD, mental health, physiology

## Abstract

**Background:**

Posttraumatic stress disorder (PTSD) is a prevalent psychiatric condition that is associated with symptoms such as hyperarousal and overreactions. Treatments for PTSD are limited to medications and in-session therapies. Assessing the way the heart responds to PTSD has shown promise in detecting and understanding the onset of symptoms.

**Objective:**

This study aimed to extract statistical and mathematical approaches that researchers can use to analyze heart rate (HR) data to understand PTSD.

**Methods:**

A scoping literature review was conducted to extract HR models. A total of 5 databases including Medical Literature Analysis and Retrieval System Online (Medline) OVID, Medline EBSCO, Cumulative Index to Nursing and Allied Health Literature (CINAHL) EBSCO, Excerpta Medica Database (Embase) Ovid, and Google Scholar were searched. Non–English language studies, as well as studies that did not analyze human data, were excluded. A total of 54 studies that met the inclusion criteria were included in this review.

**Results:**

We identified 4 categories of models: descriptive time-independent output, descriptive and time-dependent output, predictive and time-independent output, and predictive and time-dependent output. Descriptive and time-independent output models include analysis of variance and first-order exponential; the descriptive time-dependent output model includes a classical time series analysis and mixed regression. Predictive time-independent output models include machine learning methods and analysis of the HR-based fluctuation-dissipation method. Finally, predictive time-dependent output models include the time-variant method and nonlinear dynamic modeling.

**Conclusions:**

All of the identified modeling categories have relevance in PTSD, although the modeling selection is dependent on the specific goals of the study. Descriptive models are well-founded for the inference of PTSD. However, there is a need for additional studies in this area that explore a broader set of predictive models and other factors (eg, activity level) that have not been analyzed with descriptive models.

## Introduction

### Background

Posttraumatic stress disorder (PTSD) is a psychiatric condition that develops as a result of experiencing injury, severe psychological shock, and other trauma [1]. Individuals with PTSD are affected by the recall of traumatic experiences and often develop depression, anxiety, emotional instabilities, and suicidal thoughts [2]. Recent reports suggest that individuals with PTSD are about 5 times more likely to commit suicide than individuals without PTSD [3]. Approximately 10% of American women and 4% of American men experience PTSD in their lifetime [4]. PTSD is an endemic among veterans as well, affecting between 17% and 24% of veterans from recent conflicts [5].

Although an alarming number of individuals are afflicted with PTSD, there are significant barriers to care delivery [6,7]. These barriers include a shortage of qualified clinicians and understaffed mental health clinics, geographical constraints to accessing mental health facilities, financial obstacles, and cultural factors such as social stigma, and limited capabilities in objective diagnosis (currently limited to self-reported measures such as the PTSD checklist [PCL-5]) [8]. Studies have shown that self-management and factors such as positivity directly affect PTSD symptoms and ease in dealing with them [9]. Mobile health (mHealth) apps have shown promise in facilitating self-management (eg, education, mindfulness, and self-assessment) and have the potential to facilitate direct communication between people who have PTSD and their health care providers [10]. mHealth apps deployed on wearable devices (eg, smartwatches) that are equipped with an array of physiological sensors (eg, heart rate [HR]) may also enable continuous remote monitoring of signs and symptoms of PTSD. Indeed, recent efforts have shown promising applications of watch-based HR sensors to detect the onset of PTSD hyperarousal events [11].

### Objectives

Despite recent work, the extent of knowledge on the physiological reactions to PTSD and, in particular, HR is limited, and research is needed to better understand the changes in HR associated with PTSD. Few models (eg, analysis of variance [ANOVA], regression analysis) have been developed to relate changes in heart activity to disorder states. In particular, given the opportunity to collect HR data nonintrusively, it is important to use appropriate mathematical and statistical methods to ensure the accumulation of convergent knowledge in this field and to characterize and understand HR in terms of PTSD. In this paper, we document the findings from a review of the current literature on measures and models used in various domains to analyze HR data. In addition to summarizing and synthesizing the HR analysis methods, we provide an evaluation of methods for applications relevant to PTSD detection and diagnosis.

## Methods

### Search Strategy

A scoping review was conducted using the strategies outlined in the preferred reporting items for systematic reviews and meta-analyses (PRISMA) methodology [12]. The scoping review approach was selected because it is effective for knowledge evaluation and gap identification [13]. The review spanned 5 main databases: (1) Medical Literature Analysis and Retrieval System Online (Medline) OVID, (2) Medline EBSCO, (3) Cumulative Index to Nursing and Allied Health Literature (CINAHL) EBSCO, (4) Excerpta Medica Database (Embase) Ovid, and (5) Google Scholar. Search terms included heart*, pulse*, heart rate*, model*, heart beat*, and analysis*. All studies published in or after the year 2000 were included. This search was supplemented by a secondary search of cited articles in the results. The search was completed on January 15, 2020.

### Study Selection, Inclusion, and Exclusion Criteria

Abstracts were reviewed for relevance, and articles that did not discuss HR-related measures in detail and did not provide or use quantitative methods for analysis were excluded. Other exclusion criteria were non–English language articles and articles that assessed non–heart-based physiological measures such as skin conductance and blood pressure. Furthermore, studies that did not analyze human physiology were excluded. The inclusion criteria were all articles that discussed human HR analysis. Our initial search yielded 1905 results. After removing duplicate articles and checking for eligibility using Rayyan QCRI (a web app for assisting literature reviews), 270 articles were further reviewed. Out of the 270, 138 were exclusively about non–heart-based measures reactions, 67 did not focus on human physiology, and 11 had duplicated content. Of these, 54 articles from the search were included in this review based on their relevance to the topic.

**Figure 1:**
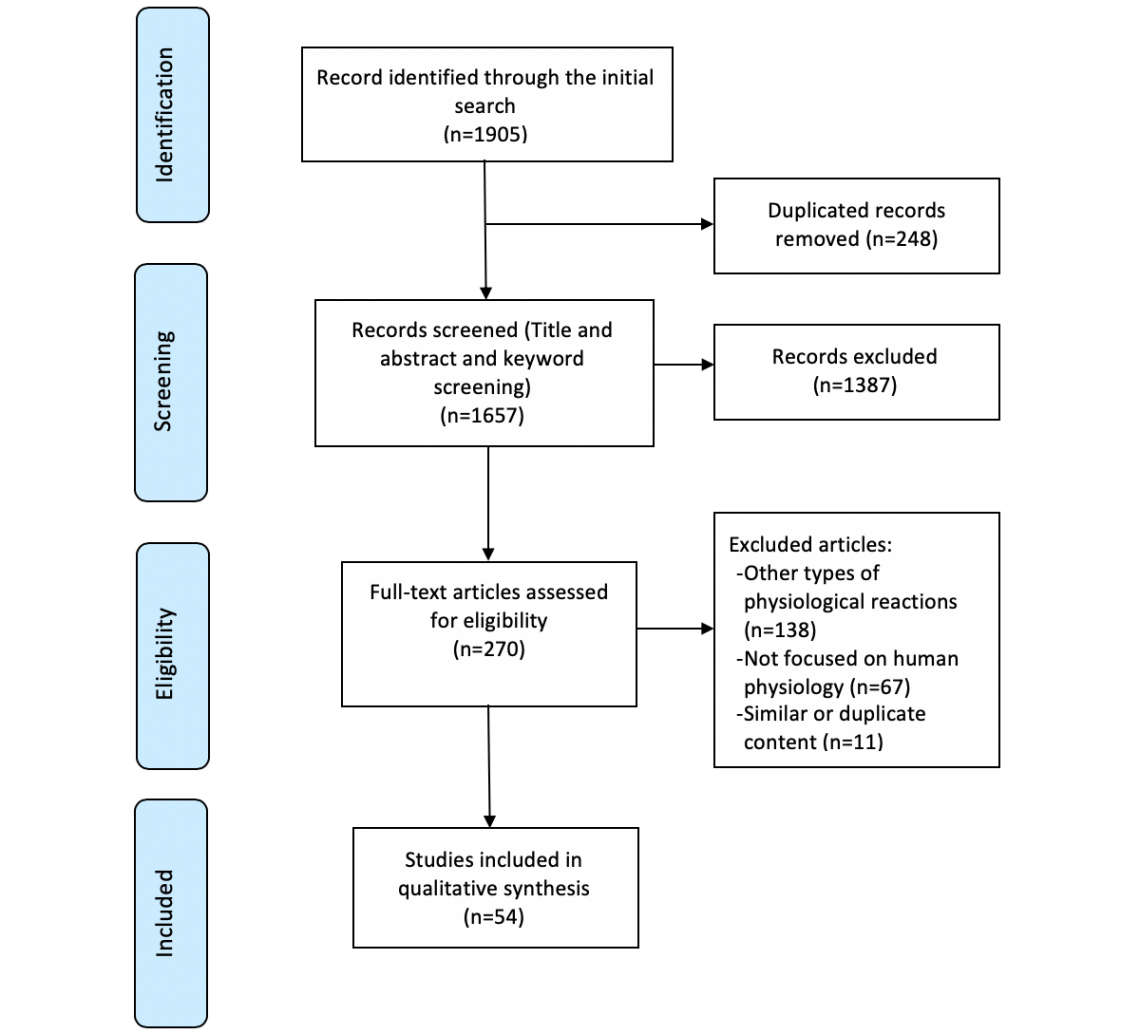
Preferred reporting items for systematic reviews and meta-analyses flow chart for the literature review.

Furthermore, the bibliography of references in each research paper was investigated thoroughly (backward search) to identify pertinent articles, and then Google Scholar searches (forward search) were conducted to find the full text. Figure 1 shows the PRISMA flow chart for the article selection process.

## Results

We listed the articles identified by the search process into 2 categories based on our synthesis: studies of the effects of PTSD on heart physiology and quantitative modeling techniques for heart data. We further partitioned studies of PTSD effects into 2 types: (1) studies that investigate the effect of PTSD on heart rate variability (HRV) and (2) studies that explore the effect of PTSD on HR. The literature on models can be further classified by the model’s focus on describing versus predicting data and the model output. These categories and subdivisions are discussed in the following sections.

### Effects of Posttraumatic Stress Disorder on Heart Rate Variability

HRV measures variations in heartbeats and is related to the electrical activity of the heart [14]. Common frequency domain analysis metrics for HRV include high frequency power (HF), low frequency power (LF), the ratio of LF to HF, coherence score (COH), root mean square of successive differences between normal heartbeats (RMSSD), and the SD of the interbeat interval of normal sinus beats (SDNN) [15-18]. LF and HF are frequency bands of HRV that tend to correlate with parasympathetic nervous system activity. LF is the frequency activity in the range of 0.04 to 0.15 Hz, and HF is the activity in the range of 0.15 to 0.4 Hz. The quantified relative intensity of these measures is referred to as power [1], and such power is obtained by applying power spectral and frequency domain analyses [19].

The reviewed articles found that PTSD causes sustained changes in the autonomic nervous system (ANS; the part of the nervous system that is responsible for regulating automated functions in the body, such as heart activity) [20]. The ANS consists of the parasympathetic nervous system (PNS), which regulates blood pressure and breathing rate during rest, and the sympathetic nervous system (SNS), which adjusts blood pressure and HR during activity. Heart activity is representative of the performance of these systems [21]. Various effects of PTSD on ANS have also been documented. Higher HR levels indicate lower HRV and are linked to increased rates of mental stress and physical activity [22,23]. PTSD, as a particular type of anxiety disorder, also disturbs HR and HRV. HRV has been studied widely in the literature to assess PTSD [18,24-26]. Evidence suggests that individuals with PTSD have lower resting HRV than individuals without PTSD when other factors (age, gender, and health level) are controlled [27]. According to the meta-review Nagpal et al [1], HF, a measure for the parasympathetic activity of ANS, is significantly lower in individuals with PTSD than in individuals without PTSD (approximately 0.6 ms^2^). However, LF, which assesses both the sympathetic and parasympathetic activity of the ANS, is slightly reduced in individuals with PTSD (approximately 0.2 ms^2^). This results in a significant increase in LF divided by HF of individuals with PTSD [1,28-30].

RMSSD and SDNN are time-domain measures of HRV. SDNN is an index of SNS activity [24]. SDNN is decreased in individuals with PTSD compared with healthy individuals (approximately 6.7 ms), showing an increase in sympathetic activity [1,31]. In addition, decreased levels of RMSSD was observed among individuals with PTSD (approximately 7.5 ms), suggesting lower vagal activity in this population [1,31].

Although an HRV analysis is common among studies of anxiety [32], some factors need to be considered when HRV measures are used. First, studies show that HRV is dependent on HR and cannot be analyzed independently to represent ANS activity [32,33]. In addition, previous research has linked high HRV to pathological conditions related to heart deficiencies [32]. For instance, diseases such as atrial fibrillation increase HRV and HR and are associated with higher mortality rates [34]. Hence, higher rates of HRV do not always indicate an abnormal mental state. Ideally, measurements should take into account patient’s comorbidities such as heart deficiencies in addition to subjective (eg, self-reported scales) and objective (eg, HRV, ECG) methods [35]. Gender, health, age, and HR also affect HRV, and they need to be considered as covariates when HRV measures are used [24]. Aging decreases HRV time-domain features such as SDNN [36,37]. HRV time-domain features increase with improved health conditions [38,39]. LF and SDNN are also lower in females than in males; however, the HF parameter of HRV is greater in women than in men [40]. Higher HR levels are also associated with decreased HRV [41] because when the heart beats faster, the beat-to-beat intervals are smaller. Other factors such as climate, job satisfaction, lifestyle, and medications can also affect HRV and should be considered as an influential factor when HRV is analyzed [42].

### Effect of Posttraumatic Stress Disorder on Heart Rate

HR is the number of heartbeats per 60 seconds. Normal HR differs among individuals based on age and gender, health level, and respiratory activity [43]. Both HR and HRV are modulated by the ANS [44]. As the SNS activates, PNS activity is suppressed; therefore, HR increases and HRV decreases [45]. As a result, there is an inverse relationship between HR and HRV [33].

PTSD can affect HR in 2 modalities: resting and fluctuation tone [1,46-48]. Studies suggest that resting HR can be between 5 and 6.6 beats higher in individuals with PTSD than in individuals without PTSD depending on the type of population (eg, veteran, civilian) [49-51]. For example, resting HR is roughly higher than 5 beats per minute in civilians with PTSD than in civilians without PTSD, and this number increases to 6.6 beats per minute in the veteran population [51,52]. In the nonresting state, evidence suggests that HR increases with exposure to PTSD stressors [1].

Another HR measure that has been investigated in terms of PTSD is HR fluctuations (changes in HR levels) in the presence of stimuli [53]. There are conflicting findings on the comparison of this measure between individuals with and without PTSD. Although a study by Roy et al [54] showed that HR changes are higher in people with PTSD than in people without PTSD, a study by Halligan et al [55] claims the opposite.

### Heart Rate Models

**Figure 2:**
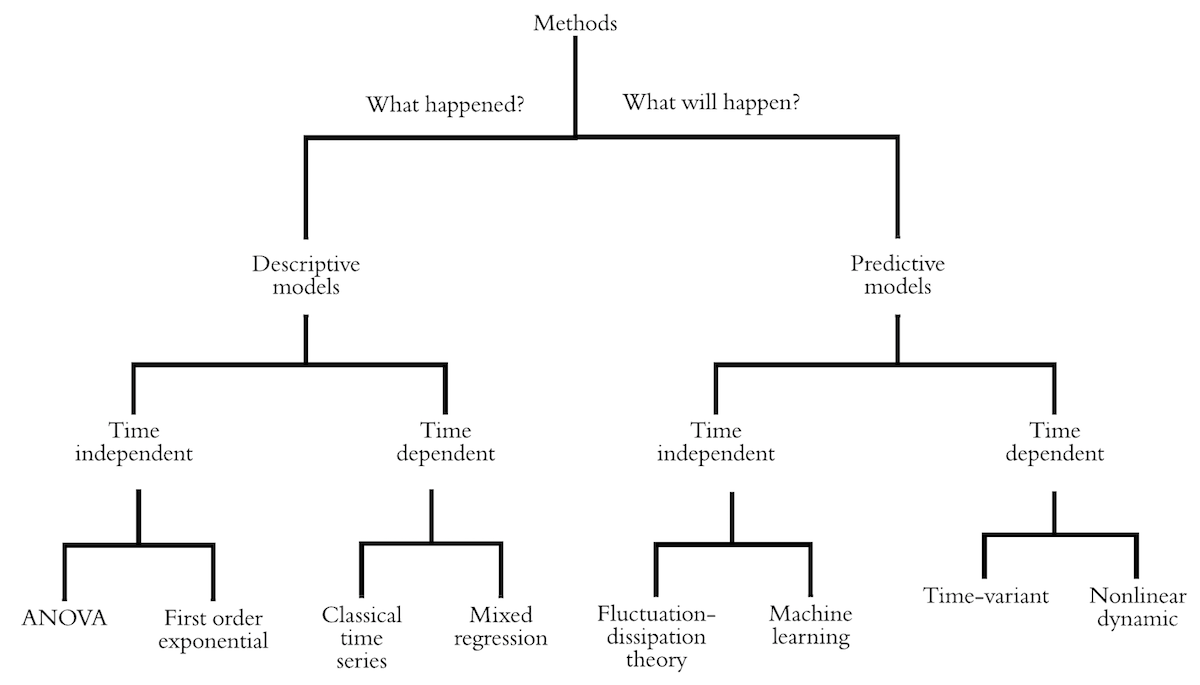
Taxonomy of heart rate analysis methods. ANOVA: analysis of variance.

On the basis of our synthesis of the existing literature, we categorized mathematical models of HR into descriptive and predictive models, both of which could provide insight relevant to understanding the psychophysiological responses to PTSD. Descriptive methods can be used to describe and make inferences about a data set, whereas predictive methods can be applied to forecast trends and patterns in the data. Predictive and descriptive models can be further characterized by their type of output—time independent or time dependent (Figure 2). Time-dependent outputs use time as one of the descriptive variables to analyze the dependent variable(s) or output(s). Time-independent output, however, does not depend on time and does not change over time. Although the models reviewed below are summarized and synthesized for relevance to PTSD-related analysis, these methods are not limited to PTSD and anxiety disorder domains.

#### Descriptive Models

##### Time-Independent Output

###### Analysis of Variance

Linear regression, and in particular ANOVA, is a statistical model used for the analysis of HR in several articles (Table 1). ANOVA can be used to compare HR trends and group means in experimental studies [56,57]. Studies have used ANOVA to account for the effectiveness of treatments in individuals with PTSD, as measured by HR [58]. Some studies chose ANOVA as their method of analysis to show that resting HR is higher in individuals with PTSD than in individuals without PTSD [57]. For example, the study by Gelpin et al [59] compared the resting HR in individuals pre-and posttreatment to measure the success of therapy sessions. Buckley et al [52] used ANOVA to compare resting HR in patients with PTSD with that of healthy controls, finding that patients with PTSD, in general, have significantly higher resting HR levels (approximately a 6 beats-per-minute difference). Although using ANOVA for the analysis of time-independent HR data is highly common, ANOVA is limited in several respects. ANOVA has strong assumptions and is ill-suited to model-dependent measures with strong temporal correlations. For instance, the independency of observations is one of the main assumptions of ANOVA; however, consecutive HR real time–based data are a highly correlative type of data. Thus, ANOVA should not be used to make time-based HR predictions [60].

###### First-Order Exponential Model

A first-order exponential model provides a function with a sustained growth or decay rate [61]. In terms of HR analysis, first-order exponential models have been used to generate a nonlinear regression model for HR based on heart rate recovery (HRR) [62]. HRR is an indicator of vagal reactivation and SNS deactivation [63].

Bartels-Ferreira et al [63] used the first-order exponential method to measure postexercise time-independent HRR based on HR decay curves. Recovering from the onset of PTSD symptoms is associated with activation of vagal tone and withdrawal of SNS activity, both of which are correlated with HRR [64]. Although this method shows promise in the assessment of HR fluctuations associated with PTSD, the reviewed literature (Table 1) examined ANS in the context of physical activity, and HR decay after activity was curve fitted by a first-order exponential function [63]. In this case, the goodness of fit was moderate (*R*^2^ was approximately 0.65), which warrants additional research. Another limitation associated with this method is that the exponential functions show erroneous patterns for very small (30-second) and very large (600-second) time windows [61]. For instance, Bartels-Ferreira et al [63] found that the least goodness of fit was for the smallest time window, which was 30 seconds (*R*^2^=0.42). Conversely, when the length of the window of time was a moderate number (approximately 360 seconds), a relatively better goodness of fit was obtained (approximately 0.69). This shows that the HRR curve fitted by first-order exponential models performs better (higher *R*^2^) when windows of times are neither too big nor too small. Table 1 shows a summary of articles that studied descriptive models with time-independent output. In this table, domain is the field of the study. Independent variables are factors that are controlled by researchers, and dependent variables are dependent on them. *Independent variables* are used to describe or classify dependent variable.

**Table 1:** Results of studies that used descriptive models with time-independent output.

Method and authors		Domain	Independent variables	Dependent variables
**ANOVA^a^**				
	Shalev et al [57]	PTSD^b^	Gender, age, HR^c^, trauma history, event security	HR
	Strath et al [65]	Physical activity	HR, oxygen intake, age, fitness	HR
	Romero-Ugalde et al [66]	Physical activity	Accelerometer, energy expenditure, HR	HR
	Khoueiry et al [67]	Medical	HR, hospitalization duration, age	HR
	Tonhajzerova et al [68]	Physiology	Resting HR, major depressive disorder	HR
**First-order exponential**				
	Bartels et al [63]	Physical activity	HR peak, resting HR, HRR^d^	HR variation

^a^ANOVA: analysis of variance.

^b^PTSD: posttraumatic stress disorder.

^c^HR: heart rate.

^d^HRR: heart rate recovery.

#### Time-Dependent Output

### Classical Time Series Analysis

Classical time series analysis is a common statistical method that can analyze time-dependent data trends by looking into linear relationships. Classical time series analysis is also a promising method for analyzing HR and HR fluctuations as these measures are time-based [69,70].

Peng et al [70] applied time series analysis to examine the long-term correlation within HR data and its relation to heart diseases such as cogestive heart failure. Using this method, the authors showed that there is some independency between beat-to-beat HR fluctuations in healthy people that does not exist in patients with cardiovascular disease. The findings further suggest that classical time series analysis is a promising direction for PTSD hyperarousal analysis because similar HR changes have been documented in patients with PTSD compared with healthy people in the presence of stimuli [71].

Beyond the analogous use case, the classical time series has several benefits compared with ANOVA. As the model explicitly considers autocorrelation, it does not require the assumption of independence of observations [72]. The models also have predictive capability and are well validated for illustrating trends and forecasting [73]. However, 1 drawback of this method is the stationary assumption (constant mean value of the series), which is not always reasonable in HR data (eg, when data are collected before and during exercise).

### Mixed Regression Model

Mixed regression analysis has been used in the literature to evaluate physiological responses to energy expenditure [74]. This type of modeling can be applied with correlated observations. Thus, it is beneficial for psychophysiology analyses that need to account for individual similarities such as gender [60]. Multiple regression typically proceeds in a stepwise process with a focus on identifying 2 main effects: the population fixed effect and the random effect. The population-fixed effect explains similarities in the dataset (for instance HR), whereas the random effect represents the differences among observations (the error term). For instance, Gee et al [75] used respiration as a random effect to estimate HR and ultimately predict episodes of bradycardia in infants. Using a mixed regression method and accounting for respiration as a covariate, in this case, has increased the accuracy of the measured HR by 11%.

The ability of mixed regression models to account for individual differences makes them an advantageous choice for modeling PTSD. Several studies have identified significant individual differences in people with PTSD [1,57,76,77]. Specifically, HR and HRV levels are significantly affected by individual differences such as age, general health, and gender [24].

This type of modeling might produce similar results to ANOVA in many cases. However, in comparison with ANOVA, mixed regression models are more effective for data sets with missing values and multiple random effects [78]. This is important as in real-world and naturalistic studies, data sets with high rates of missing values are common and can be challenging to deal with [79]. Table 2 shows a comparison of time-dependent output methods.

**Table 2:** Results from studies that used descriptive models with time-dependent output.

Method and authors			Domain		Independent variables		Dependent variable
**Classical time series**							
	Chen et al [69]	Health care (patient data)		HR^a^, resting HR		Heartbeat	
	Kazmi et al [33]	Physiology		HR, HRV^b^, time		HR	
	Zakeri et al [80]	Physical activity		HR, energy expenditure, accelerometer, age		Energy expenditure	
	Peng et al [70]	Medical		HR, heartbeat, time		HR	
**Mixed regression**							
	Gee et al [75]	Biomedical		HR, heartbeat, respiration, time		HR	
	Bonomi et al [81]	Physical activity		HR, energy expenditure, photoplethysmography, accelerometer		HR	
	Xu et al [82]	Physical activity		HR, energy expenditure, different training paradigms, age, height, weight		Energy expenditure	

^a^HR: heart rate.

^b^HRV: heart rate variability.

#### Predictive Models

##### Time-Independent Output

###### Machine Learning Methods

Machine learning methods refer to a set of training and predictive algorithms that use data to learn complex trends associated with labels (eg, symptom presence) in a data set. Machine learning analysis is a multiple-step process consisting of dividing a data set into training and testing data (or leveraging resampling techniques such as cross-validation), developing a model from the training data, and evaluating the model on the testing data. This approach is advantageous relative to approaches that use all of the data for training a model (eg, ANOVA) and approximate metrics to evaluate generalizability (eg, adjusted *R*^2^). Furthermore, the ability of machine learning algorithms to identify complex patterns in data sets make them a promising approach for analyzing physiological data that are often noisy.

The success of applying machine learning methods depends on the data used to train and evaluate the algorithm. Machine learning algorithms typically require large training sets—several thousand observations—and they implicitly assume that the data and associated labels are of equal quality. In cases where the data are noisy, or labels are unreliable, machine learning training algorithms may fail to converge to a generalizable solution. Furthermore, if the training data examples are biased (eg, nonrepresentative population samples), the machine learning algorithms trained on the data may also be similarly biased. It is often difficult to identify these issues through standard training and testing processes of machine learning algorithms; thus, machine learning analyses should be accompanied by descriptive analyses to obtain a better understanding of the data and potential errors or bias [83].

Most of the reviewed studies used HRV, along with machine learning algorithms to predict stress levels in individuals [84-86]. Machine learning studies evaluating HR have primarily focused on energy expenditure [87,88]. An exception is McDonald et al [11] who evaluated several machine learning algorithms—neural networks, decision trees, support vector machines, convolutional neural networks, and random forests—to predict the onset of PTSD symptoms in the veteran population. This study used HR data with a 1 Hz frequency (1 observation per second) as the input of these algorithms. Although the raw 1 Hz data were used to train the neural network–based models, additional feature generation and selection was performed before training the decision tree, support vector machine, and random forest algorithms. This feature generation identified linear trends, Fourier transforms, and change quantiles as relevant features for the detection of the onset of PTSD symptoms. Among all machine learning methods, support vector machines, and random forest algorithms performed best (ie, had the highest area under the receiver operating characteristic curve (ROC) 0.67). Although machine learning shows promise for the inferential analysis of HR data for PTSD research, explaining the purpose of machine learning components may be difficult, and often predictive results have a limited rational explanation [89].

###### Fluctuation-Dissipation Theory

The fluctuation-dissipation theory (FDT) is a common approach in thermodynamics that is used to predict system behavior by breaking the system responses into small forces [90]. This theorem, which follows thermodynamic rules, can model the HRR after stress moments.

Chen et al [91] used FDT to predict patients’ HR reactions to prespontaneous and postspontaneous breathing trial treatment. They used this method to divide the system (in this case, the treatment process) into different phases, including pretreatment, midtreatment, and posttreatment. After breaking the entire treatment process to these small phases, each phase was modeled separately. The reactions to treatments in each phase were modeled using HRR measures. All models were then combined to create the final comprehensive model. Chen et al [91] found that thermodynamic rules can also model the HR response after stress moments. This is because of the similar effect of stress and spontaneous breathing trials on organs (a common clinical procedure used to assess the ventilation performance of patients). These researchers suggest dividing the system into prestress and poststress moments, modeling each phase, and finally assembling a model for final prediction. They further suggest that the HRR extracted from this type of modeling can be used to personalize care as HR can be remotely monitored through noninvasive hospital devices.

In terms of mathematical concepts, this type of modeling has a powerful predictive capability by grouping individuals and therefore minimizing the error rate [91]. This approach requires significantly less data than other methods, such as time-variant modeling of HR. Hence, it enables researchers to include more variables in their model. Moreover, Chen et al [91] claim that although models that use Gaussian functions have around 65% error rate to predict patients’ response to spontaneous breathing trial, implementing FDT decreases this error rate by over 10%. Therefore, this approach provides more accurate results than methods that use Gaussian functions, such as some machine learning algorithms (eg, adaptive neuro-fuzzy inference system [ANFIS]). A potential reason for this could be that the system is broken down into smaller pieces, where each part has its own specific and defining features. However, in ANFIS, the system was considered as a whole, and a set of features was defined for the entire system overlooking dissimilarities within the system. In addition, unlike most statistical approaches that make assumptions about the data, this method is assumption-free and is considered more robust to assumptions (eg, normality of residuals, independency of measurements). Despite its promising application to the analysis of HR and the lack of restrictive assumptions, FDT is computationally intense. This means that the model needs a high level of proficiency in understanding the mathematics and statistics behind FDT. Especially, in comparison with approaches such as ANOVA, classical time series, and mixed regression, using this approach requires higher levels of domain knowledge, for example, studies in machine learning and FDT methods (Table 3).

**Table 3:** Results from example studies that used predictive models with time-independent output.

Method and authors		Domain		Independent variables		Dependent variable	
**Machine learning**							
	Kolus et al [87]	Biomedical (energy expenditure)		HR^a^, oxygen consumption, work rate		Work rate	
	McDonald et al [11]	PTSD^b^		HR, subjective stress moments		Stress moment	
	Healey et al [86]	Driving		HR, HRV^c^, skin conductance, muscle activity, muscle tension, breathing rate		To detect stress	
	Kolus et al [88]	Physical activity		HR, maximum HR, oxygen consumption, body type, work rate		Work rate	
	Zhang et al [92]	Physical activity		HR, body attitude information, body movement		HR	
**Fluctuation-dissipation theory**							
	Chen et al [91]	Health care		HR recovery, blood pressure, instantaneous HR		HR	

^a^HR: heart rate.

^b^PTSD: posttraumatic stress disorder.

^c^HRV: heart rate variability.

#### Time-Dependent Output

### Time-Variant Modeling

Time-variant modeling is a mathematical approach used to analyze time-dependent data sets and provide a time-dependent output. Time-variant models of HR can generate HRR measures in real time. Some studies suggest that measuring HRR in real time can especially help assess arousals and arousability in different individuals in response to mental stressors [93]. This shows promise for PTSD research given its potential to enable the comparison between the effect of internal stimuli (stressors generated through memory) and external stimuli (stressors generated from the environment) on the arousability of patients with PTSD.

Although time-variant modeling has been replicated in the literature and has shown promise in analyzing HR data [33,94], it is computationally intense. The process of solving the equations within the model includes defining multiplex matrices for each variable, which is time and space consuming. Moreover, time-variant modeling requires large data sets of HF (eg, 100 Hz) HR data, which is often not feasible for real-time data collection instruments such as wearable devices that record continuous data for large windows of time (eg, more than 30 min).

### Nonlinear Dynamic Modeling

Nonlinear dynamic modeling of HR consists of depicting HR as the output of a nonlinear dynamic system [95].

Nonlinear dynamic modeling of HR can be a promising method to assess arousal patterns by measuring SNS activity [96]. Hence, this approach may be useful for analyzing PTSD hyperarousal patterns as they are associated with SNS activity. Despite the advantages of this model, it requires high-frequency HR data (eg, 100 Hz) or even instantaneous HR [96]. Instantaneous HR is an HR measure derived from HRV, which is different from raw HR measured by wearable devices. Instantaneous HR can be extracted by multiplying RR intervals (the time between two consecutive R waves of the HRV signal) by 60 and needs to be measured at an HF (>250 Hz), whereas smartwatches collect HR data with a much lower frequency (<5 Hz) [96].

This model accounts for the natural nonlinearity and time-dependent features of HR data. In addition, the learnability and predictability of this method can help detect the onset of symptoms in patients with PTSD. A limitation of this method for characterizing PTSD aspects is the assumption of invertibility [97]. This assumption indicates that all the variable matrices used in equations are required to be invertible. In many cases, and mainly in nonlaboratory settings, this assumption cannot be met [97]. Moreover, these methods are relatively slow and more computationally intense compared with other methods such as machine learning (for both training and testing the model) because they involve solving multiple complex mathematical equations [66]. Table 4 shows examples of predictive models with time-dependent output.

**Table 4:** Results from studies that used predictive models with time-dependent output.

Method and authors		Domain		Independent variables		Dependent variable	
**Time variant**							
	Lefever et al [94]		Sports science—biomedical		HR^a^, participants’ input power, road gradient,		HRV^b^
	Olufsen et al [98]		Biology, health care		HR, resting HR, blood pressure		HR regulations
**Nonlinear dynamic**							
	Chen et al [69]		Health care (patient data)		Resting HR, arterial blood pressure, HR, HRV		Heart beat
	Kazmi et al [33]		Biophysics		Human normal sinus rhythm, human congestive heart rate failure		HRV (they look at the correlation)

^a^HR: heart rate.

^b^HRV: heart rate variability.

## Discussion

### Descriptive Framework Based on the Summary of Findings

We categorized the methods used to analyze HR data into 2 categories: descriptive and predictive. In the context of PTSD, descriptive models may be used to characterize PTSD triggers and the factors that affect their occurrence, whereas predictive models may be useful to predict PTSD onset to facilitate timely intervention. The extracted models provide methods for evaluating, describing, comparing, interpreting, and understanding patterns in the HR data. However, interpreting the data in a meaningful way depends on the specific objectives of the study. The data at hand can be analyzed with one or many of the reviewed models based on the goal of the study and the assumptions of the models. Each model corresponds to a distinct type of output and different interpretations of the data with different assumptions. On the basis of the process of data collection, the number of observations, and variables in the data, researchers might choose one or a combination of models provided. Table 5 provides a framework for choosing a model based on the limitations, assumptions, and features of each model and the data at hand. Furthermore, Table 5 presents the articles that used a specific method.

**Table 5:** Descriptive framework for heart rate–related analysis methods extracted from the literature.

Model		Assumptions	Features	Limitations	Cases
**Descriptive, time-independent output**					
	ANOVA^a^	Normal distribution of residuals Constant variance of populations Independence and identically distributed observations	Capable of comparing groups and looking at trends Computationally simple	Restrictive assumptions Type 1 error Just applicable to linear analysis	[47,52-54,57-59, 65-68,99-102]
	A first-order exponential model	Continuous observations Observations should be identical (eg, no age, gender difference) Environmental effects are constant	Easy to apply and learn Gives higher weights to recent observations	Not repeated in studies Higher error rates than classical time series and mixed regression Does not show trends Not accurate for very small and very large windows of time	[63]
**Descriptive, time-dependent output**					
	Classical time series analysis	Stationary observations (constant mean values of series)	Advantageous for analyzing time-based trends Does not require independence of data points Used in the literature to analyze cardiovascular disease Includes linear and nonlinear analysis	Requires stationary data sets	[33,69,70,80]
	Mixed regression model	Normality of residuals distribution	Accounts for differences between individuals (eg, age, gender) Can be used for analyzing repeated measures Can be applied to non-normal data	Cannot be used for nonlinear models	[50,66,67,75, 80-82,103-107]
**Predictive, time-independent output**					
	Machine learning methods	Limited dependencies of the observations (each machine learning algorithm has its assumptions that need to be checked)	Proactive algorithm (can be used for action-reaction type of data sets) Powerful predictive method Rapid analysis prediction, and processing Simplifies time-intensive computations	Can over fit or under fit data Cannot be applied to data sets with highly dependent variables The process has little rational explanation	[11,86-88,92,108-110]
	Fluctuation-dissipation theory	Equilibrium system (the system and observations are not changing)	Powerful predictive capability Does not have restrictive assumptions such as normality of residuals Significantly less data needed compared with a general data fitting approach	Computationally intense Time consuming	[70,91,111]
**Predictive, time-dependent output**					
	Time-variant modeling	Requires big data sets with high-frequency data points (more than 60 Hz)	Can be used to describe data as well as forecasting the future	Computationally intense Slow process	[33,93,94,96, 98,112-116]
	Nonlinear dynamic modeling	Invertible matrices	Very accurate Replicated multiple times in studies	Computationally intense Slow process Requires invertible matrices that is not always feasible in naturalistic settings	[33,66,96,98,104, 112,113,116-121]

^a^ANOVA: analysis of variance.

### Fit Assessment

Fit assessment can be conducted to examine the efficiency of each method in modeling a specific dataset. Fit assessment is especially promising for comparing different methods if they are applied to the same data set. However, considering the wide range of applicable fit indices, researchers might struggle to compare them. In the category of descriptive models, *R*^2^ and adjusted *R*^2^ are the main indices of fit assessment. *R*^2^ indicates the degree of variation in the dependent variable caused by the independent variable(s). Adjusted *R*^2^ is a revised version of *R*^2^ that accounts for the number of independent variables in a model [122]. Generally, adjusted *R*^2^ is more promising than *R*^2^ as it is more robust to overfitting [122]. In the prediction methods category, a variety of measures other than *R*^2^ and adjusted *R*^2^ were used to assess the quality of fit. Some of these measures include sensitivity, specificity, accuracy, and area under the ROC curve (AUC)-ROC. Sensitivity is the number of true-positives divided by the total number of observations, and specificity is the number of true-negatives divided by the total number of observations [123]. Accuracy is the number of true predictions divided by the total number of predictions. The error rate is 1 minus the accuracy or the number of wrong detections divided by the total number of observations [124]. Finally, AUC-ROC is a curve that plots the true-positive rate (*Y* axis) versus the false-positive rate (*X* axis) to measure the performance of the model. It is important to bear in mind that fit indices are data dependent; therefore, comparisons are the best made by fitting multiple models to the same data set.

In the statistical analysis of data in the PTSD domain, fit assessments have been used to show the efficiency of the results. For instance, McDonald et al [11] used ROC curves along with accuracy to show that random forest works better than other machine learning methods to predict hyperarousal moments in people with PTSD. Shalev et al [125] used sensitivity and specificity to predict the development of PTSD based on their instant responses to trauma. Bartels et al [63] applied adjusted *R*^2^ to assess the goodness of fit for their proposed exponential model. Examples of fit adjustments are summarized in Table 6.

**Table 6:** Examples of fit assessment for different methods used in studies.

Study	Method	Variables	Fit measure
Strath et al [65]	ANOVA^a^	HR^b^, oxygen intake, age, fitness	*R*^2^=0.87
Zakeri et al [80]	Classical time series	HR, energy expenditure, accelerometer, age	*R*^2^=0.84
McDonald et al [11]	Machine learning	HR, subjective stress moments	Area under receiver operating characteristics curve=0.67
Healey et al [86]	Machine learning	HR, HRV^c^, skin conductance, muscle activity, muscle tension, breathing rate	Accuracy=97%
Chen et al [91]	Fluctuation-dissipation theory	HR recovery, blood pressure, instantaneous HR	Error rate=25%
Chen et al [66]	Nonlinear dynamic	Resting HR, arterial blood pressure, HR, HRV	Sensitivity=0.941; predictability=0.988

^a^ANOVA: analysis of variance.

^b^HR: heart rate.

^c^HRV: heart rate variability.

### Methodological Considerations for Heart Rate Assessments

The models identified in this review represent several promising directions for future exploration, but they also illustrate a hidden complexity in the use of HR data as model input. HR is impacted by individual characteristics including age, sex, health, resting HR, respiration, and lifestyle [24]. The maximum HR typically decreases with age. Females have higher HR levels than men [126]. Athletes have lower HR levels than sedentary people [127]. Resting HR is lower in more active people, and lower resting HRs result in lower HR levels [128]. As the respiratory system affects heart activity, studies suggest that incorporating respiration as a factor in HR models improves HR estimation significantly [78]. Lifestyle such as smoking habits affects HR as well; people who smoke have a higher HR than nonsmokers [129].

Beyond these general characteristics, it is important to consider the type of physical activity in the analysis. Physical activity significantly affects HR [130], where high-intensity activities such as running and cycling affect HR differently from low-intensity activities such as sitting and lying down [99]. Concerns regarding activity were common in the reviewed studies, particularly in the energy expenditure domain [131]. Green et al [131] suggested that body acceleration is a reliable indicator of physical activity and should be included in all analyses as a covariate or constraint. Although activity is directly related to energy expenditure outcomes, it is also relevant for studies investigating stress. Whereas some of the reviewed studies on stress included body acceleration in their analysis [100], many neglected this factor [46,132].

### Heart Rate Assessments in Anxiety Domains

HR data have been widely investigated in the domains of physical activity and energy expenditure. Although there are some differences between the effects of mental stress on HR and the effects of physical activity on HR, there are many similarities that make these domains connected. Physical activity affects SNS performance in the short term and PNS performance in the long term [133]. As a result, HR increases during physical activities (due to SNS activation), and resting HR is lower in athletes who have higher rates of physical activity (because of PNS performance) [133].

Similarly, in terms of mental stress, whereas acute stress or immediate response to stressors activates SNS, chronic stress increases vagal and parasympathetic activity [134]. These similarities enable researchers in mental stress domains to employ models and pathways that are extracted in physical activity domains. For instance, one main measure that is used broadly to examine energy expenditure is HRR. This measure is an accepted indicator of SNS deactivation and PNS activation. Recovering from acute stress and arousability is also associated with the withdrawal of SNS and activation of PNS. As a result, HRR can be a proper measure to be considered in studies that examine acute stress.

### Limitations

This scoping review attempted to include all articles that analyzed HR; however, it is still likely that some were overlooked. Furthermore, the authors categorized the HR models based on their own synthesis of the literature and relevance to PTSD. These models can be listed and categorized in a variety of ways, such as deterministic versus stochastic.

Another limitation of this review is that although the identified models have been applied across various domains (eg, energy expenditure and general stress prediction), to our knowledge, only 2 papers [11,57] directly applied these methods to data from patients diagnosed with PTSD. In particular, only 1 study [11] used a predictive approach in the PTSD domain. Other studies were primarily limited to linear descriptive statistics such as the *t* test or ANOVA [60,65–67]. These methods are valid for making inferences about PTSD and comparing their effects on HR among different groups. However, there is a need for additional studies in this area that explore a broader set of predictive models and other factors (eg, activity level) that have not been analyzed with descriptive models.

Beyond the specific application of these models to PTSD, there are several more general challenges. The reviewed research often proceeded independently, with few links between the various studies. This diversity makes comparisons across studies difficult. Studies have used different data sets with different variables based on individual goals. Furthermore, the reviewed work often focused on testing 1 specific model rather than a broad comparison. Often critical details, such as the model and parameter selection process, were not reported in the articles. Another critical detail often not addressed in the reviewed studies was the mismatch between the model requirements and the sampling rates, which may result in conditions such as overfitting [135].

Collectively, these limitations suggest a need for substantial additional work in modeling the relationship between HR and PTSD. Future studies should consider comparisons between several models, analyze or explicitly discuss decisions made throughout the modeling process, and comprehensively document their HR data collection. As future studies are conducted that enact these criteria, the utility of the modeling approaches identified here will become clearer, and the path to more effective PTSD treatments will become more attainable.

### Conclusions

The goals of this review were to identify and characterize quantitative HR models for relevant applications in PTSD. One of the gaps in this area is the absence of a framework that researchers can use before, during, and after their data collection to choose a method to analyze HR data. In this regard, we developed a descriptive framework that can be used to determine the method to apply to HR data to achieve more efficient results. We identified 4 broad categories of methods: descriptive time-independent output, descriptive time-dependent output, predictive time-independent output, and predictive time-dependent output. Descriptive time-independent output models include ANOVA and first-order exponential, whereas descriptive time-dependent output models include classical time series analysis and mixed regression. Predictive time-independent output models include machine learning methods and analysis of HR-based FDT. Finally, predictive time-independent output models include the time-variant method and nonlinear dynamic modeling.

All of the identified modeling categories have relevance in PTSD, although modeling selection is highly dependent on the specific goals of the modeler. For instance, one might use ANOVA to examine the differences in resting HR in individuals with PTSD versus without PTSD [54].

## Abbreviations

ANFIS: adaptive neuro-fuzzy inference system
ANOVA: analysis of variance
ANS: autonomic nervous system
AUC-ROC: area under the receiver operating characteristics curve
COH: coherence score
FDT: fluctuation-dissipation theory
HF: high frequency power
HR: heart rate
HRR: heart rate response
HRV: heart rate variability
LF: low frequency power
mHealth: mobile health
PNS: parasympathetic nervous system
PRISMA: preferred reporting items for systematic reviews and meta-analyses
PTSD: posttraumatic stress disorder
RMSSD: root mean square of successive differences between normal heart beats
SDNN: SD of the interbeat interval of normal sinus beats
SNS: sympathetic nervous system

## References

[1] Gleichauf ML (2013). Meta-analysis of heart rate variability as a psychophysiological indicator of posttraumatic stress disorder. J Trauma Treat.

[2] Spivak B, Segal M, Mester R, Weizman A (1998). Lateral preference in post-traumatic stress disorder. Psychol Med.

[3] LeBouthillier DM, McMillan KA, Thibodeau MA, Asmundson GJ (2015). Types and number of traumas associated with suicidal ideation and suicide attempts in PTSD: findings from a US nationally representative sample. J Trauma Stress.

[4] Resnick HS, Kilpatrick DG, Dansky BS, Saunders BE, Best CL (1993). Prevalence of civilian trauma and posttraumatic stress disorder in a representative national sample of women. J Consult Clin Psychol.

[5] Richardson LK, Frueh BC, Acierno R (2010). Prevalence estimates of combat-related post-traumatic stress disorder: critical review. Aust N Z J Psychiatry.

[6] Moon J, Smith A, Sasangohar F, Benzer JK, Kum H (2017). A descriptive model of the current PTSD care system: identifying opportunities for improvement. Proc Int Symp Hum Factors Ergon Health Care.

[7] Reisman M (2016). PTSD treatment for veterans: what's working, what's new, and what's next. Pharm Ther.

[8] Rodriguez-Paras C, Tippey K, Brown E, Sasangohar F, Creech S, Kum H, Lawley M, Benzer JK (2017). Posttraumatic stress disorder and mobile health: app investigation and scoping literature review. JMIR Mhealth Uhealth.

[9] Khusid MA, Vythilingam M (2016). The emerging role of mindfulness meditation as effective self-management strategy, part 1: clinical implications for depression, post-traumatic stress disorder, and anxiety. Mil Med.

[10] Galea S, Basham K, Culpepper L, Davidson J, Foa E, Kizer K, Koenen K, Leslie D, McCormick R, Milad M (2012). Treatment for Posttraumatic Stress Disorder in Military and Veteran Populations: Initial Assessment.

[11] McDonald AD, Sasangohar F, Jatav A, Rao AH (2019). Continuous monitoring and detection of post-traumatic stress disorder (PTSD) triggers among veterans: a supervised machine learning approach. IISE Trans Healthc Syst Eng.

[12] Moher D, Liberati A, Tetzlaff J, Altman DG, PRISMA Group (2009). Preferred reporting items for systematic reviews and meta-analyses: the PRISMA statement. Ann Intern Med.

[13] Tricco AC, Lillie E, Zarin W, O'Brien K, Colquhoun H, Kastner M, Levac D, Ng C, Sharpe JP, Wilson K, Kenny M, Warren R, Wilson C, Stelfox HT, Straus SE (2016). A scoping review on the conduct and reporting of scoping reviews. BMC Med Res Methodol.

[14] Acharya UR, Joseph KP, Kannathal N, Lim CM, Suri JS (2006). Heart rate variability: a review. Med Biol Eng Comput.

[15] Cohen H, Benjamin J, Geva AB, Matar MA, Kaplan Z, Kotler M (2000). Autonomic dysregulation in panic disorder and in post-traumatic stress disorder: application of power spectrum analysis of heart rate variability at rest and in response to recollection of trauma or panic attacks. Psychiatry Res.

[16] Ginsberg J, Ayers E, Burriss L, Powell D (2008). Discriminative delay Pavlovian eyeblink conditioning in veterans with and without posttraumatic stress disorder. J Anxiety Disord.

[17] Ginzburg K, Ein-Dor T, Solomon Z (2010). Comorbidity of posttraumatic stress disorder, anxiety and depression: a 20-year longitudinal study of war veterans. J Affect Disord.

[18] Tan G, Dao TK, Farmer L, Sutherland RJ, Gevirtz R (2011). Heart rate variability (HRV) and posttraumatic stress disorder (PTSD): a pilot study. Appl Psychophysiol Biofeedback.

[19] Kamath M, Fallen E (1993). Power spectral analysis of heart rate variability: a noninvasive signature of cardiac autonomic function. Crit Rev Biomed Eng.

[20] Prins A, Kaloupek D, Keane T, Friedman MJ, Charney DS, Deutch AY (1995). Psychophysiological evidence for autonomic arousal and startle in traumatized adult populations. Neurobiological and Clinical Consequences of Stress: From Normal Adaptation to Post-traumatic Stress Disorder.

[21] Hynynen E, Uusitalo A, Konttinen N, Rusko H (2006). Heart rate variability during night sleep and after awakening in overtrained athletes. Med Sci Sports Exerc.

[22] Buchheit M, Simon C, Charloux A, Doutreleau S, Piquard F, Brandenberger G (2006). Relationship between very high physical activity energy expenditure, heart rate variability and self-estimate of health status in middle-aged individuals. Int J Sports Med.

[23] Weber CS, Thayer JF, Rudat M, Sharma AM, Perschel FH, Buchholz K, Deter HC (2008). Salt-sensitive men show reduced heart rate variability, lower norepinephrine and enhanced cortisol during mental stress. J Hum Hypertens.

[24] Shaffer F, Ginsberg JP (2017). An overview of heart rate variability metrics and norms. Front Public Health.

[25] Wahbeh H, Oken BS (2013). Peak high-frequency HRV and peak alpha frequency higher in PTSD. Appl Psychophysiol Biofeedback.

[26] Pyne JM, Constans JI, Wiederhold MD, Gibson DP, Kimbrell T, Kramer TL, Pitcock JA, Han X, Williams DK, Chartrand D, Gevirtz RN, Spira J, Wiederhold BK, McCraty R, McCune TR (2016). Heart rate variability: pre-deployment predictor of post-deployment PTSD symptoms. Biol Psychol.

[27] Gillie BL, Thayer JF (2014). Individual differences in resting heart rate variability and cognitive control in posttraumatic stress disorder. Front Psychol.

[28] Mellman TA, Pigeon WR, Nowell PD, Nolan B (2007). Relationships between REM sleep findings and PTSD symptoms during the early aftermath of trauma. J Trauma Stress.

[29] Lee SM, Han H, Jang K, Huh S, Huh HJ, Joo J, Chae J (2018). Heart rate variability associated with posttraumatic stress disorder in victims' families of Sewol ferry disaster. Psychiatry Res.

[30] Minassian A, Maihofer AX, Baker DG, Nievergelt CM, Geyer MA, Risbrough VB, Marine Resiliency Study Team (2015). Association of predeployment heart rate variability with risk of postdeployment posttraumatic stress disorder in active-duty marines. JAMA Psychiatry.

[31] Park JE, Lee JY, Kang S, Choi JH, Kim TY, So HS, Yoon I (2017). Heart rate variability of chronic posttraumatic stress disorder in the Korean veterans. Psychiatry Res.

[32] Monfredi O, Lyashkov AE, Johnsen A, Inada S, Schneider H, Wang R, Nirmalan M, Wisloff U, Maltsev VA, Lakatta EG, Zhang H, Boyett MR (2014). Biophysical characterization of the underappreciated and important relationship between heart rate variability and heart rate. Hypertension.

[33] Kazmi SZ, Zhang H, Aziz W, Monfredi O, Abbas SA, Shah SA, Kazmi SS, Butt WH (2016). Inverse correlation between heart rate variability and heart rate demonstrated by linear and nonlinear analysis. PLoS One.

[34] Stein PK, Domitrovich PP, Hui N, Rautaharju P, Gottdiener J (2005). Sometimes higher heart rate variability is not better heart rate variability: results of graphical and nonlinear analyses. J Cardiovasc Electrophysiol.

[35] Centre for Urban Design and Mental Health.

[36] Bonnemeier H, Richardt G, Potratz J, Wiegand UK, Brandes A, Kluge N, Katus HA (2003). Circadian profile of cardiac autonomic nervous modulation in healthy subjects: differing effects of aging and gender on heart rate variability. J Cardiovasc Electrophysiol.

[37] Almeida-Santos MA, Barreto-Filho JA, Oliveira JL, Reis FP, da Cunha OC, Sousa AC (2016). Aging, heart rate variability and patterns of autonomic regulation of the heart. Arch Gerontol Geriatr.

[38] Agelink MW, Boz C, Ullrich H, Andrich J (2002). Relationship between major depression and heart rate variability. Clinical consequences and implications for antidepressive treatment. Psychiatry Res.

[39] Liao D, Cai J, Brancati FL, Folsom A, Barnes RW, Tyroler HA, Heiss G (1995). Association of vagal tone with serum insulin, glucose, and diabetes mellitus-the ARIC study. Diabetes Res Clin Pract.

[40] Koenig J, Thayer JF (2016). Sex differences in healthy human heart rate variability: a meta-analysis. Neurosci Biobehav Rev.

[41] Zhang D, Shen X, Qi X (2016). Resting heart rate and all-cause and cardiovascular mortality in the general population: a meta-analysis. Can Med Assoc J.

[42] Sammito S, Böckelmann I (2016). Factors influencing heart rate variability. Int Cardiovasc Forum J.

[43] Umetani K, Singer DH, McCraty R, Atkinson M (1998). Twenty-four hour time domain heart rate variability and heart rate: relations to age and gender over nine decades. J Am Coll Cardiol.

[44] Albright TD, Jessell TM, Kandel ER, Posner MI (2000). Neural science. Cell.

[45] Mathias C, Bannister R (2013). Autonomic Failure: A Textbook of Clinical Disorders of the Autonomic Nervous System.

[46] Shalev AY, Sahar T, Freedman S, Peri T, Glick N, Brandes D, Orr SP, Pitman RK (1998). A prospective study of heart rate response following trauma and the subsequent development of posttraumatic stress disorder. Arch Gen Psychiatry.

[47] Pole N, Cumberbatch E, Taylor WM, Metzler TJ, Marmar CR, Neylan TC (2005). Comparisons between high and low peritraumatic dissociators in cardiovascular and emotional activity while remembering trauma. J Trauma Dissociation.

[48] Bryant RA, Creamer M, O'Donnell M, Silove D, McFarlane AC (2008). A multisite study of initial respiration rate and heart rate as predictors of posttraumatic stress disorder. J Clin Psychiatry.

[49] Beckham JC, Feldman ME, Barefoot JC, Fairbank JA, Helms MJ, Haney TL, Hertzberg MA, Moore SD, Davidson JR (2000). Ambulatory cardiovascular activity in Vietnam combat veterans with and without posttraumatic stress disorder. J Consult Clin Psychol.

[50] Kannel WB, Kannel C, Paffenbarger RS, Cupples L (1987). Heart rate and cardiovascular mortality: the Framingham study. Am Heart J.

[51] Woodward SH, Arsenault NJ, Voelker K, Nguyen T, Lynch J, Skultety K, Mozer E, Leskin GA, Sheikh JI (2009). Autonomic activation during sleep in posttraumatic stress disorder and panic: a mattress actigraphic study. Biol Psychiatry.

[52] Buckley TC, Holohan D, Greif JL, Bedard M, Suvak M (2004). Twenty-four-hour ambulatory assessment of heart rate and blood pressure in chronic PTSD and non-PTSD veterans. J Trauma Stress.

[53] Orr SP, Pitman RK, Lasko NB, Herz LR (1993). Psychophysiological assessment of posttraumatic stress disorder imagery in world war II and Korean combat veterans. J Abnorm Psychol.

[54] Roy M, Costanzo M, Jovanovic T, Leaman S, Taylor P, Norrholm S, Rizzo A (2013). Heart rate response to fear conditioning and virtual reality in subthreshold PTSD. Stud Health Technol Inform.

[55] Halligan SL, Michael T, Wilhelm FH, Clark DM, Ehlers A (2006). Reduced heart rate responding to trauma reliving in trauma survivors with PTSD: correlates and consequences. J Trauma Stress.

[56] Tabachnick BG, Fidell LS (2011). Experimental Designs Using ANOVA.

[57] Shalev AY, Freedman S, Peri T, Brandes D, Sahar T, Orr SP, Pitman RK (1998). Prospective study of posttraumatic stress disorder and depression following trauma. Am J Psychiatry.

[58] Foa EB, Rothbaum BO, Riggs DS, Murdock TB (1991). Treatment of posttraumatic stress disorder in rape victims: a comparison between cognitive-behavioral procedures and counseling. J Consult Clin Psychol.

[59] Gelpin E, Bonne O, Peri T, Brandes D, Shalev A (1996). Treatment of recent trauma survivors with benzodiazepines: a prospective study. J Clin Psychiatry.

[60] Cacioppo J, Tassinary L, Berntson G (2007). Handbook of Psychophysiology.

[61] Hardy GH (2016). Properties of logarithmico-exponential functions. Proc Lond Math Soc.

[62] Marquardt DW (1963). An algorithm for least-squares estimation of nonlinear parameters. J Soc Ind Appl Math.

[63] Bartels-Ferreira R, de Sousa ED, Trevizani GA, Silva LP, Nakamura FY, Forjaz CL, Lima JR, Peçanha T (2015). Can a first-order exponential decay model fit heart rate recovery after resistance exercise?. Clin Physiol Funct Imaging.

[64] Lipov E (2013). Post traumatic stress disorder (PTSD) as an over activation of sympathetic nervous system: an alternative view. J Trauma Treat.

[65] Strath SJ, Swartz AM, Bassett DR, O'Brien WL, King GA, Ainsworth BE (2000). Evaluation of heart rate as a method for assessing moderate intensity physical activity. Med Sci Sports Exerc.

[66] Romero-Ugalde HM, Garnotel M, Doron M, Jallon P, Charpentier G, Franc S, Huneker E, Simon C, Bonnet S (2017). An original piecewise model for computing energy expenditure from accelerometer and heart rate signals. Physiol Meas.

[67] Khoueiry Z, Roubille C, Nagot N, Lattuca B, Piot C, Leclercq F, Delseny D, Busseuil D, Gervasoni R, Davy J, Pasquié JL, Cransac F, Sportouch-Dukhan C, Macia J, Cung T, Massin F, Cade S, Cristol J, Barrère-Lemaire S, Roubille F (2012). Could heart rate play a role in pericardial inflammation?. Med Hypotheses.

[68] Tonhajzerova I, Ondrejka I, Chladekova L, Farsky I, Visnovcova Z, Calkovska A, Jurko A, Javorka M (2012). Heart rate time irreversibility is impaired in adolescent major depression. Prog Neuropsychopharmacol Biol Psychiatry.

[69] Chen H, Erol Y, Shen E, Russell S (2016). Probabilistic model-based approach for heart beat detection. Physiol Meas.

[70] Peng C, Havlin S, Stanley HE, Goldberger AL (1995). Quantification of scaling exponents and crossover phenomena in nonstationary heartbeat time series. Chaos.

[71] Cohen H, Kotler M, Matar MA, Kaplan Z, Loewenthal U, Miodownik H, Cassuto Y (1998). Analysis of heart rate variability in posttraumatic stress disorder patients in response to a trauma-related reminder. Biol Psychiatry.

[72] Kantz H, Schreiber T (2004). Nonlinear Time Series Analysis.

[73] Montgomery DC, Johnson LA, Gardiner JS (1990). Forecasting And Time Series Analysis.

[74] Galwey NW (2014). Introduction to Mixed Modelling: Beyond Regression and Analysis of Variance.

[75] Gee A, Barbieri R, Paydarfar D, Indic P (2016). Improving heart rate estimation in preterm infants with bivariate point process analysis of heart rate and respiration. Conf Proc IEEE Eng Med Biol Soc.

[76] Boscarino JA (2008). A prospective study of PTSD and early-age heart disease mortality among Vietnam veterans: implications for surveillance and prevention. Psychosom Med.

[77] Kassam-Adams N, Garcia-España JF, Fein JA, Winston FK (2005). Heart rate and posttraumatic stress in injured children. Arch Gen Psychiatry.

[78] Darlington RB (1990). Regression and Linear Models.

[79] Greenacre M (2017). Correspondence Analysis in Practice.

[80] Zakeri I, Adolph A, Puyau M, Vohra F, Butte N (2013). Cross-sectional time series and multivariate adaptive regression splines models using accelerometry and heart rate predict energy expenditure of preschoolers. J Nutr.

[81] Bonomi A, Goldenberg S, Papini G, Kraal J, Stut W, Sartor F, Kemps H (2015). Predicting Energy Expenditure From Photo-Plethysmographic Measurements of Heart Rate Under Beta Blocker Therapy: Data Driven Personalization Strategies Based on Mixed Models. Proceedings of the 37th Annual International Conference of the IEEE Engineering in Medicine and Biology Society.

[82] Xu Z, Zong C, Jafari R (2015). Constructing energy expenditure regression model using heart rate with reduced training time. Conf Proc IEEE Eng Med Biol Soc.

[83] James G, Witten D, Hastie T, Tibshirani R (2013). An Introduction to Statistical Learning: with Applications in R.

[84] Sano A, Picard R (2013). Stress Recognition Using Wearable Sensors and Mobile Phones. Proceedings of the Humaine Association Conference on Affective Computing and Intelligent Interaction.

[85] Thayer JF, Ahs F, Fredrikson M, Sollers JJ, Wager TD (2012). A meta-analysis of heart rate variability and neuroimaging studies: implications for heart rate variability as a marker of stress and health. Neurosci Biobehav Rev.

[86] Healey J, Picard R (2005). Detecting stress during real-world driving tasks using physiological sensors. IEEE Trans Intell Transport Syst.

[87] Kolus A, Dubé PA, Imbeau D, Labib R, Dubeau D (2014). Estimating oxygen consumption from heart rate using adaptive neuro-fuzzy inference system and analytical approaches. Appl Ergon.

[88] Kolus A, Imbeau D, Dubé PA, Dubeau D (2016). Classifying work rate from heart rate measurements using an adaptive neuro-fuzzy inference system. Appl Ergon.

[89] Michie D, Spiegelhalter D, Taylor C (1994). Machine Learning, Neural and Statistical Classification.

[90] Kubo R (2002). The fluctuation-dissipation theorem. Rep Prog Phys.

[91] Chen M, Niestemski LR, Prevost R, McRae M, Cholleti S, Najarro G, Buchman TG, Deem MW (2013). Prediction of heart rate response to conclusion of the spontaneous breathing trial by fluctuation dissipation theory. Phys Biol.

[92] Zhang Y, Chen W, Su SW, Celler B (2012). Nonlinear modelling and control for heart rate response to exercise. Int J Bioinform Res Appl.

[93] Roger D, Jamieson J (1988). Individual differences in delayed heart-rate recovery following stress: the role of extraversion, neuroticism and emotional control. Personal Individ Differ.

[94] Lefever J, Berckmans D, Aerts J (2014). Time-variant modelling of heart rate responses to exercise intensity during road cycling. Eur J Sport Sci.

[95] Haber R, Unbehauen H (1990). Structure identification of nonlinear dynamic systems—a survey on input/output approaches. Automatica.

[96] Valenza G, Lanata A, Scilingo EP (2012). The role of nonlinear dynamics in affective valence and arousal recognition. IEEE Trans Affective Comput.

[97] Langford J, Salakhutdinov R, Zhang T (2009). Learning Nonlinear Dynamic Models. Proceedings of the 26th Annual International Conference on Machine Learning.

[98] Olufsen MS, Ottesen JT (2013). A practical approach to parameter estimation applied to model predicting heart rate regulation. J Math Biol.

[99] Boulay MR, Simoneau JA, Lortie G, Bouchard C (1997). Monitoring high-intensity endurance exercise with heart rate and thresholds. Med Sci Sports Exerc.

[100] Vrijkotte TG, van Doornen LJ, de Geus EJ (2000). Effects of work stress on ambulatory blood pressure, heart rate, and heart rate variability. Hypertension.

[101] Feng J, Huang Z, Zhou C, Ye X (2018). Study of continuous blood pressure estimation based on pulse transit time, heart rate and photoplethysmography-derived hemodynamic covariates. Australas Phys Eng Sci Med.

[102] Champéroux P, Fesler P, Judé S, Richard S, le Guennec JY, Thireau J (2018). High-frequency autonomic modulation: a new model for analysis of autonomic cardiac control. Br J Pharmacol.

[103] Diderichsen PM, Cox E, Martin SW, Cleton A, Ribbing J (2013). Predicted heart rate effect of inhaled PF-00610355, a long acting β-adrenoceptor agonist, in volunteers and patients with chronic obstructive pulmonary disease. Br J Clin Pharmacol.

[104] Hoyer D, Nowack S, Bauer S, Tetschke F, Rudolph A, Wallwitz U, Jaenicke F, Heinicke E, Götz T, Huonker R, Witte OW, Schleussner E, Schneider U (2013). Fetal development of complex autonomic control evaluated from multiscale heart rate patterns. Am J Physiol Regul Integr Comp Physiol.

[105] Alrefaie M, Summerskill S, Jackon T (2019). In a heart beat: using driver's physiological changes to determine the quality of a takeover in highly automated vehicles. Accid Anal Prev.

[106] Oliveira M, Marçôa R, Moutinho J, Oliveira P, Ladeira I, Lima R, Guimarães M (2019). Reference equations for the 6-minute walk distance in healthy Portuguese subjects 18-70 years old. Pulmonology.

[107] Sartipy U, Savarese G, Dahlström U, Fu M, Lund LH (2019). Association of heart rate with mortality in sinus rhythm and atrial fibrillation in heart failure with preserved ejection fraction. Eur J Heart Fail.

[108] Ni J, Muhlstein L, McAuley J (2019). Modeling Heart Rate and Activity Data for Personalized Fitness Recommendation. Proceedings of the The World Wide Web Conference.

[109] Signorini MG, Pini N, Malovini A, Bellazzi R, Magenes G (2020). Integrating machine learning techniques and physiology based heart rate features for antepartum fetal monitoring. Comput Methods Programs Biomed.

[110] Chaudhuri T, Soh YC, Li H, Xie L (2020). Machine learning driven personal comfort prediction by wearable sensing of pulse rate and skin temperature. Build Environ Elsevier.

[111] Lu Y, Burykin A, Deem MW, Buchman TG (2009). Predicting clinical physiology: a Markov chain model of heart rate recovery after spontaneous breathing trials in mechanically ventilated patients. J Crit Care.

[112] Valenza G, Citi L, Barbieri R (2014). Estimation of instantaneous complex dynamics through Lyapunov exponents: a study on heartbeat dynamics. PLoS One.

[113] Ferrer E, Helm JL (2013). Dynamical systems modeling of physiological coregulation in dyadic interactions. Int J Psychophysiol.

[114] Valenza G, Lanatà A, Scilingo EP (2012). Oscillations of heart rate and respiration synchronize during affective visual stimulation. IEEE Trans Inf Technol Biomed.

[115] Zazula D (2012). Optimization of Heartbeat Detection Based on Clustering and Multimethod Approach. Proceedings of the Annual International Conference of the IEEE Engineering in Medicine and Biology Society.

[116] Echeverría JC, Álvarez-Ramírez J, Peña MA, Rodríguez E, Gaitán MJ, González-Camarena R (2012). Fractal and nonlinear changes in the long-term baseline fluctuations of fetal heart rate. Med Eng Phys.

[117] Park Y, Ryu K, Shim S, Hoh J, Park M (2013). Comparison of fetal heart rate patterns using nonlinear dynamics in breech versus cephalic presentation at term. Early Hum Dev.

[118] Scalzi S, Tomei P, Verrelli CM (2012). Nonlinear control techniques for the heart rate regulation in treadmill exercises. IEEE Trans Biomed Eng.

[119] Cheng T, Savkin A, Celler B, Su S, Wang L (2008). Nonlinear modeling and control of human heart rate response during exercise with various work load intensities. IEEE Trans Biomed Eng.

[120] Mazzoleni MJ, Battaglini CL, Martin KJ, Coffman EM, Mann BP (2016). Modeling and predicting heart rate dynamics across a broad range of transient exercise intensities during cycling. Sports Eng.

[121] Zakynthinaki MS (2015). Modelling heart rate kinetics. PLoS One.

[122] Gelman A, Hill J (2006). Data Analysis Using Regression and Multilevel/Hierarchical Models.

[123] Harrington P (2012). Machine Learning In Action.

[124] D'Agostino RB (1986). Goodness-of-Fit-Techniques.

[125] Shalev AY, Peri T, Canetti L, Schreiber S (1996). Predictors of PTSD in injured trauma survivors: a prospective study. Am J Psychiatry.

[126] Magder SA (2012). The ups and downs of heart rate. Crit Care Med.

[127] Lester M, Sheffield L, Trammell P, Reeves T (1968). The effect of age and athletic training on the maximal heart rate during muscular exercise. Am Heart J.

[128] Sacknoff DM, Gleim GW, Stachenfeld N, Coplan NL (1994). Effect of athletic training on heart rate variability. Am Heart J.

[129] Dietrich DF, Schwartz J, Schindler C, Gaspoz J, Barthélémy JC, Tschopp J, Roche F, von Eckardstein A, Brändli O, Leuenberger P, Gold DR, Ackermann-Liebrich U, SAPALDIA-Team (2007). Effects of passive smoking on heart rate variability, heart rate and blood pressure: an observational study. Int J Epidemiol.

[130] Freedson PS, Miller K (2000). Objective monitoring of physical activity using motion sensors and heart rate. Res Q Exerc Sport.

[131] Green JA, Halsey LG, Wilson RP, Frappell PB (2009). Estimating energy expenditure of animals using the accelerometry technique: activity, inactivity and comparison with the heart-rate technique. J Exp Biol.

[132] Taelman J, Vandeput S, Spaepen A, Van HS (2008). Influence of Mental Stress on Heart Rate and Heart Rate Variability. Proceedings of the 4th European Conference of the International Federation for Medical and Biological Engineering.

[133] Pagani M, Somers V, Furlan R, Dell'Orto S, Conway J, Baselli G, Cerutti S, Sleight P, Malliani A (1988). Changes in autonomic regulation induced by physical training in mild hypertension. Hypertension.

[134] McCarty R, Horwatt K, Konarska M (1988). Chronic stress and sympathetic-adrenal medullary responsiveness. Soc Sci Med.

[135] Chen H, Simpson D, Ying Z (2000). Infill asymptotics for a stochastic process model with measurement error. Stat Sin.

